# Ischemia/reperfusion-activated ferroptosis in the early stage triggers excessive inflammation to aggregate lung injury in rats

**DOI:** 10.3389/fmed.2023.1181286

**Published:** 2023-06-23

**Authors:** Xiujie Liu, Binhui Pan, Xiaoting Wang, Junpeng Xu, Xinyu Wang, Zhengyang Song, Eryao Zhang, Fangyan Wang, Wantie Wang

**Affiliations:** ^1^School of Basic Medical Science, Wenzhou Medical University, Wenzhou, China; ^2^Institute of Ischemia/Reperfusion Injury, Wenzhou Medical University, Wenzhou, China; ^3^Nephrology Department, Wenzhou Central Hospital, Wenzhou, China; ^4^Department of Gastroenterology, The Second Affiliated Hospital and Yuying Children's Hospital of Wenzhou Medical University, Wenzhou, China

**Keywords:** ferroptosis, lung, ischemia/reperfusion injury, inflammation cytokines, rat

## Abstract

**Objective:**

Lung ischemia/reperfusion injury (LIRI) is a clinical syndrome of acute lung injury that occurs after lung transplantation or remote organ ischemia. Ferroptosis and inflammation are involved in the pathogenesis of LIRI according to the results of several studies on animal models. However, the interactive mechanisms between ferroptosis and inflammation contributing to LIRI remain unclear.

**Methods:**

HE staining and indicators of oxidative stress were used to evaluated the lung injury. The reactive oxygen species (ROS) level was examined by DHE staining. The quantitative Real-time PCR (qRT-PCR) and western blot analysis were employed to detect the level of inflammation and ferroptosis, and deferoxamine (DFO) was used to assess the importance of ferroptosis in LIRI and its effect on inflammation.

**Results:**

In the present study, the link of ferroptosis with inflammation was evaluated at reperfusion 30-, 60- and 180-minute time points, respectively. As the results at reperfusion 30-minute point shown, the pro-ferroptotic indicators, especially cyclooxygenase (COX)-2 and acyl-CoA synthetase long-chain family member 4 (ACSL4), were upregulated while the anti-ferroptotic factors glutathione peroxidase 4 (GPX4), cystine-glumate antiporter (XCT) and ferritin heavy chain (FTH1) were downregulated. Meanwhile, the increased level of interleukin (IL)-6, tumor necrosis factor alpha (TNF-α) and IL-1β were observed beginning at reperfusion 60-minute point but mostly activated at reperfusion 180-minute point. Furthermore, deferoxamine (DFO) was employed to block ferroptosis, which can alleviate lung injury. Expectedly, the survival rate of rats was increased and the lung injury was mitigated containing the improvement of type II alveolar cells ultrastructure and ROS production. In addition, at the reperfusion 180-minute point, the inflammation was observed to be dramatically inhibited after DFO administration as verified by IL-6, TNF-α and IL-1β detection.

**Conclusion:**

These findings suggest that ischemia/reperfusion-activated ferroptosis plays an important role as the trigger for inflammation to further deteriorate lung damages. Inhibiting ferroptosis may have therapeutic potential for LIRI in clinical practice.

## 1. Introduction

Lung ischemia/reperfusion injury (LIRI) is a common risk factor for adverse outcomes occurring when oxygen supply to the lung has been compromised, followed by a period of reperfusion (1–3). The accumulation of lipid reactive oxygen species (ROS) induced by ischemia/reperfusion (IR) and excessive inflammation is considered the main pathogenesis for exacerbating lung tissue damages (4, 5). However, traditional antioxidative and anti-inflammatory properties are unable to completely abrogate the subsequent LIRI. Therefore, it is urgent to explore the underlying mechanisms and targeted treatments for LIRI.

Ferroptosis has been recently identified as an iron- and lipid hydroperoxide-dependent non-apoptotic cell death in numerous clinical pathologies such as intestinal IR injury, myocardial infarction, and renal failure (6, 7). It has been reported that the acyl-CoA synthetase long-chain family member (ACSL4) located in the lipid membrane initiates ferroptosis (8). Some core genes related to the lipid peroxide removal system such as cystine/glutamic acid transporter (XCT) and glutathione peroxidase 4 (GPX4) have been also found to be involved in the suppression of ferroptosis (9, 10). GPX4 is known for its ability to reduce lipid hydroperoxides and other reactive oxygen species (ROS) using glutathione (GSH) as an electron donor. When GPX4 reacts with lipid hydroperoxides, GSH provides the reducing power necessary to convert the hydroperoxide into its corresponding alcohol. This reaction also regenerates the active form of GPX4, allowing it to continue to scavenge ROS and protect the cell from oxidative damage. As evidence shows, lung IR increased the tissue iron content and lipid peroxidation accumulation, along with key protein (GPX4 and ACSL4) expression alteration during reperfusion. Moreover, inhibition of ferroptosis mitigated ferroptotic damage in IR-induced lung injury by reducing lipid peroxidation and increasing the glutathione and GPX4 levels, suggesting that ferroptosis is correlated with LIRI (11). However, the specific role of ferroptosis in LIRI needs further exploration.

Excessive inflammation, another key contributor to LIRI, has been reported to be triggered by necroptosis in several animal models (12–14); however, in fact, ferroptosis is recognized as more immunogenic than necroptosis due to potentiating a series of inflammatory reactions (15). It is well-known that arachidonic acid (AA) is the main component of cell membrane lipids, and AA is metabolized as a precursor of bioactive pro-inflammatory mediators mainly through the cyclooxygenase (COX) and lipoxygenase (LOX) pathways, which are also core pathways for ACSL4, contributing to ferroptosis. In particular, ferroptosis inhibitors have been shown to have anti-inflammatory effects in models of neurological disorders, intracerebral hemorrhage, and acute kidney injury (16–18). IL-6 and TNF-α, playing a key role in the inflammatory reactions through upregulating cytokines, cyclooxygenase, adhesion molecules, and chemokines, were reported to promote the ERK pathway in different organ IR models (19–21). However, the interaction between ferroptosis and inflammation needs further investigation in LIRI.

In this study, we sought to elucidate the specific role of ferroptosis and the interaction between ferroptosis and inflammation in LIRI. To figure out the underlying mechanism, we formulated three different reperfusion points to investigate both ferroptotic and inflammatory indicators and found that key protein GPX4 and ACSL4 expression systems were altered at the 30-min reperfusion point, while interleukin (IL)-6 and tumor necrosis factor-alpha (TNF-α) were upregulated at the 180-min reperfusion point. Furthermore, inhibition of ferroptosis can ameliorate lung injury through inflammation depression. Evidenced by the improvement of lung functions in lung damage, we hypothesize that ferroptosis triggers excessive inflammation in LIRI, and inhibition of ferroptosis would serve as a promising treatment for acute lung diseases.

## 2. Methods

### 2.1. Rat model of lung IR injury

Male Sprague–Dawley (SD) rats weighing 220–270 g were purchased from Weitonglihua (Beijing, China), and the rats were fed standard food and water and were acclimated to the environment before use. They were anesthetized and subjected to IR according to the protocols approved by the Animal Care and Use Committee of the University of Wenzhou Medical University (wydw2023-0099).

A total of 60 male SD rats were randomly divided into five groups, including the sham operation group (control group), 30-min lung ischemia–reperfusion (IR) group (IR_30_ group), 60-min lung IR group (IR_60_ group), 180-min lung IR group (IR_180_ group), and deferoxamine (DFO) pretreatment group (IR_30_+DFO and IR_180_+DFO group). Rats in the sham operation group did not undergo other procedures, except for opening the left chest. The left hilum of rats in the IR group was clamped with a non-invasive vascular clamp to establish an ischemic model. After 30 min, the vascular clamp was released, and the rats underwent reperfusion for 30, 60, and 180 min. As for rats in the IR_30_+DFO group and IR_180_+DFO group, 100 mg/kg of DFO was intraperitoneally injected 1 h before clamping the left hilar (22). Each rat was injected with 500 μl of DFO solution using physiological saline as a solvent, while the control rats were injected with an equivalent volume of physiological saline, and the rest are the same as the IR_30_ or IR_180_ group.

### 2.2. Survival experiment

A total of 30 male SD rats were randomly divided into three groups, including the control group, IR group, and IR + DFO group. Rats in the control group were opened to the left chest for 30 min and then sutured. The left hilar of rats in the IR group were clamped for 30 min and then sutured. As for rats in the DFO pretreatment group, 100 mg/kg of DFO was intraperitoneally injected 1 h before clamping the left hilar, and the rest are the same as the IR group. All the rats were kept warm on an electric blanket.

### 2.3. The wet–dry weight ratio experiment

After rats were sacrificed, part of the lungs was cut and weighed as the wet weight. Then, all the lungs were put in a constant temperature oven for 48 h and weighed as the dry weight.

### 2.4. Hematoxylin–eosin (HE) staining

The lungs were retrieved from indicated animals, and sections were prepared for HE staining. The staining was performed using the standard protocol. The general histopathology examination is under a light microscope (Olympus, Tokyo, Japan).

### 2.5. Detection of ROS fluorescence levels

Dihydroethidium (DHE) was used to interact with intracellular superoxide anions to create ethidium and 2-hydroxyethidium bromide, a bright red fluorescent complex that may be used to measure the concentration of superoxide anions by measuring the intensity of the fluorescence. DHE (10 mmol/L, Beyotime Institute of Biotechnology, Shanghai, China) was incubated with frozen sections that were sectioned on a cryostat microtome (Leica, Wetzlar, Germany), 5 μm thick, and mounted onto glass slides (CITOTEST, Jiangsu, China), at 37°C, in the dark. The slices mounted by Antifade Solution were examined under an orthographic microscope after being rinsed three times in PBS.

### 2.6. Measurement of malonaldehyde (MDA), glutathione (GSH), and Fe

Malondialdehyde (MDA) in the degradation products of lipid peroxide can be condensed with thiobarbituric acid (TBA) to form a red product with a maximum absorption peak at 532 nm. The substrate of this method is thiobarbituric acid. According to the manufacturer's instructions, lung samples were homogenized with 0.9% NaCl and centrifuged to extract the supernatant. MDA, GSH, and Fe were quantified in accordance with the detection assays' directions (A006-2-1, A003-1, and A039-2-1, Nanjing Jiancheng Bioengineering Institute, Nanjing, China). MDA, GSH, and Fe levels were standardized to the level of total protein.

### 2.7. Enzyme-linked immunosorbent assay (ELISA) detection of the level of IL-6 and TNF-α in the lung

ELISA kits were purchased from Elabscience (Wuhan, China), and the level of IL-6 (E-EL-R0015c, Elabscience) and TNF-α (E-EL-R2856c, Elabscience) detection was detected following the instruction provided.

### 2.8. Transmission electron microscope (TEM) analysis

The rats were sacrificed after reperfusion, and the lung was excised and rinsed with precooled PBS (pH 7.4). A portion of the lung was incubated in 2.5% glutaraldehyde for the whole night. With a vibratome, 50-um thick slices of the lung sample were sliced. Then, 1% osmium tetroxide was used to postfix the targeted lung for 1 h; then, the tissue was dehydrated in a series of graded ethanol and embedded in epoxy resin. The polymerization was performed at 80°C for 24 h. Next, 100-nm thick ultrathin sections were cut, stained with uranyl acetate and lead citrate, and observed under a JEM2000EX TEM (JEOL, Tokyo, Japan). Five fields were randomly selected for each sample to examine mitochondria with ferroptosis features.

### 2.9. Western blot analysis

Total protein samples were extracted from tissues using RIPA lysis buffer (Yamei, Shanghai, China). Protein concentrations were determined using a BCA protein detection kit (Beyotime Institute of Biotechnology, Shanghai, China). Proteins were separated using 10% SDS-PAGE and transferred to polyvinylidene fluoride membranes. Membranes were blocked in 5% skimmed milk and then incubated with the primary antibodies ACSL4 (ab155282, Abcam, 1:10,000), transferrin receptor 1 (TFR1) (A5865, ABclonal, 1:1,000), ferritin heavy chain (FTH1) (ab65080, Abcam, 1:1,000), XCT (Abcam, ab175186, 1:5000), GPX4 (Abcam, ab125066, 1:5000), and GAPDH (0494-1-AP, Proteintech,1:1,000) at 4°C overnight. After washing three times with tris-buffered saline, the membranes were incubated with appropriate anti-rabbit or anti-mouse secondary antibodies at room temperature for 1 h. Imprinting was observed using chemiluminescence (Yamei, Shanghai, China) and an Odyssey imaging system (Li-Cor-Biosciences, NE).

### 2.10. Quantitative real-time PCR analysis

Total RNA was extracted with TRIzol reagent (Yamei, Shanghai, China) from the lung tissue and reverse-transcribed to cDNA using a kit (Vazyme, Nanjing, China). The cDNA obtained was subjected to PCR using primers designed for COX-2, solute carrier family 39, member 14 (SLC39A14), ACSL4, GPX4, IL-6, IL-1β, TNF-α, and caspase3. The primer sequences are shown in Supplementary Table 1. Gene expression was determined using the SYBR Green kit (Vazyme, Nanjing, China), according to the instructions. All the results were normalized against β-actin expression using the Thermal Cycler Dice Real Time system (TaKaRa Company, Japan).

### 2.11. Statistical analysis

GraphPad Prism version 9.0 (GraphPad Software, San Diego, CA) was used for statistical treatment. Experimental data were shown as the mean ± SD. Two-tailed unpaired Student's *t*-test and one-way ANOVA with Tukey's correction were used for all comparisons of rat-related experiments. A *P*-value of <0.05 was considered significant. The sample distribution was determined using a Kolmogorov–Smirnov normality test.

## 3. Results

### 3.1. Ferroptosis is activated by ischemia–reperfusion at an early stage in LIRI

**Figure 1:**
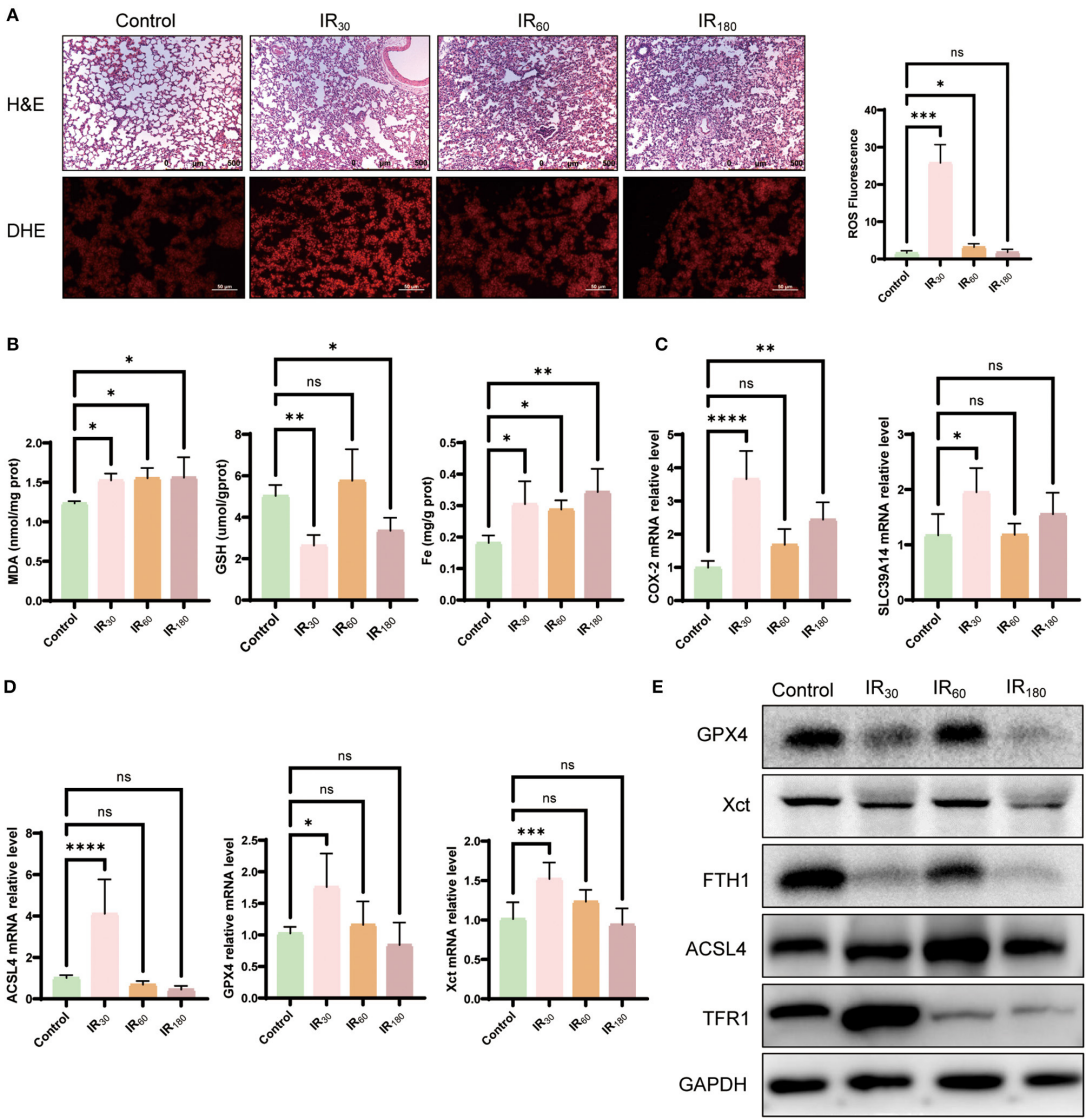
Ferroptosis is activated by ischemia–reperfusion at an early stage in LIRI. **(A)** HE and DHE staining of the lung (*n* = 3/group). **(B)** Levels of MDA, GSH, and Fe in the lung tissue (*n* = 5/group). **(C)** qRT-PCR analysis of COX-2 and SLC39A14 in the lung (*n* = 5/group). **(D)** qRT-PCR analysis of ACSL4, GPX4, and XCT (*n* = 5/group). **(E)** Western blot analysis of GPX4, XST, FTH1, ACSL4, and TFR1 in the lung (*n* = 3/group). Data are expressed as mean ± standard deviation. A one-way ANOVA was used to analyze statistical differences; *P < 0.05, **P < 0.01, ***P < 0.001, and ****P < 0.0001.

Oxidative stress is one of the main pathological mechanisms in LIRI that can induce excessive lipid peroxidation, which is also a characteristic of ferroptosis (23). Therefore, to evaluate ferroptosis sensitivity after reperfusion in the lung, we determined the level of ROS under reperfusion conditions. As shown in Figure 1A, DHE staining has been employed to assess the level of ROS induced after reperfusion at different times. Compared with the control group, the ROS level increased at the 30-min reperfusion point and decreased at the 60- and 180-min reperfusion points in IR group, which strongly suggested that oxidative stress may play a pivotal role in the early stage of LIRI, particularly at the 30-min reperfusion point. Meanwhile, HE staining showed that IR caused the disruption of lung structure, alveolar damage, and red blood cell aggregation. Similar to the above results, MDA, another essential factor for ferroptosis execution, showed a significant increase, while the reduction of the GSH level and increased Fe cytoplasmic accumulation were observed in the early phase (Figure 1B). In addition, the mRNA level of key ferroptotic factors COX-2, SLC39A14, ACSL4, GPX4, and XCT was upregulated at the early reperfusion stage (Figures 1C, D). Meanwhile, Western blot analysis showed that these anti-ferroptotic proteins such as GPX4, XCT, and FTH1 were downregulated, while pro-ferroptotic proteins ACSL4 and TFR1 were upregulated at the early phase after reperfusion (Figure 1E). These findings indicate that ferroptosis induced by IR occurs at an early stage.

### 3.2. Inflammation erupts following ferroptosis in LIRI

**Figure 2:**
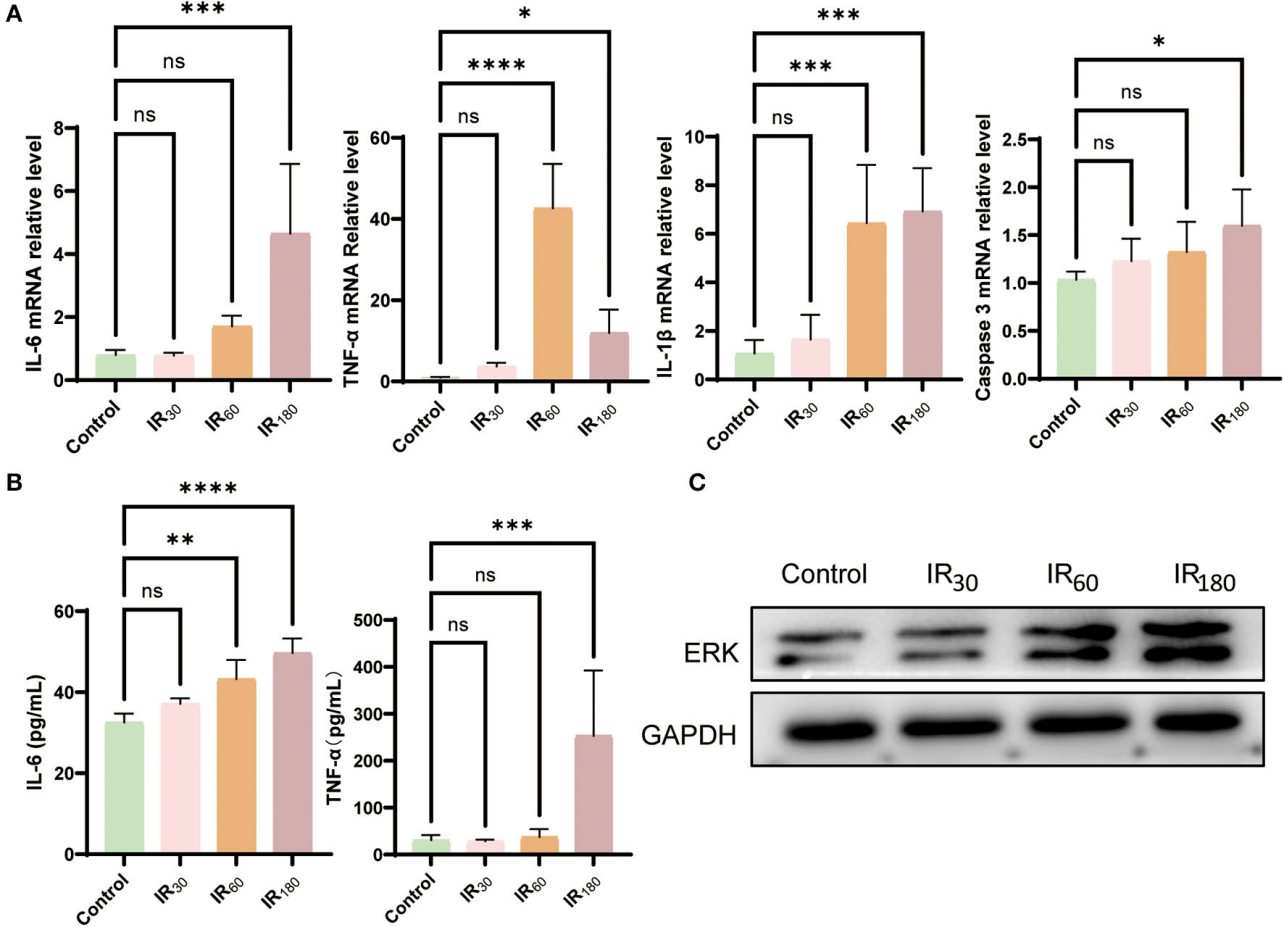
Inflammation erupts following ferroptosis in LIRI. **(A)** qRT-PCR analysis of IL-6, TNF-α, IL-1β, and caspase 3 in the lung (*n* = 5/group). **(B)** ELISA detection of IL-6 and TNF-α in the lung (*n* = 5/group). **(C)** Western blot analysis of total ERK in the lung (*n* = 3/group). Data are expressed as mean ± standard deviation. A one-way ANOVA was used to analyze statistical differences; *P < 0.05, **P < 0.01, ***P < 0.001, and ****P < 0.0001.

Since ferroptosis is likely to be a trigger of inflammation, we further investigated some inflammation cytokines occurring at different reperfusion stages in LIRI. As results shown in Figure 2A, the gene expression of inflammatory cytokines IL-6, TNF-α, and IL-1β were remarkably increased during prolonged reperfusion along with the upregulated level of caspase 3. To reconfirm the above results, ELISA detection was used to examine the level of IL-6 and TNF-α. As expected, the level of the two key inflammatory cytokines was upregulated at the 180-min reperfusion point (Figure 2B). Furthermore, Western blot analysis of total ERK showed that the ERK increased along with the extension of reperfusion time (Figure 2C). As a result, excessive inflammation erupts following ferroptosis in LIRI.

### 3.3. DFO attenuates lung damage by inhibiting ferroptosis in LIRI

**Figure 3:**
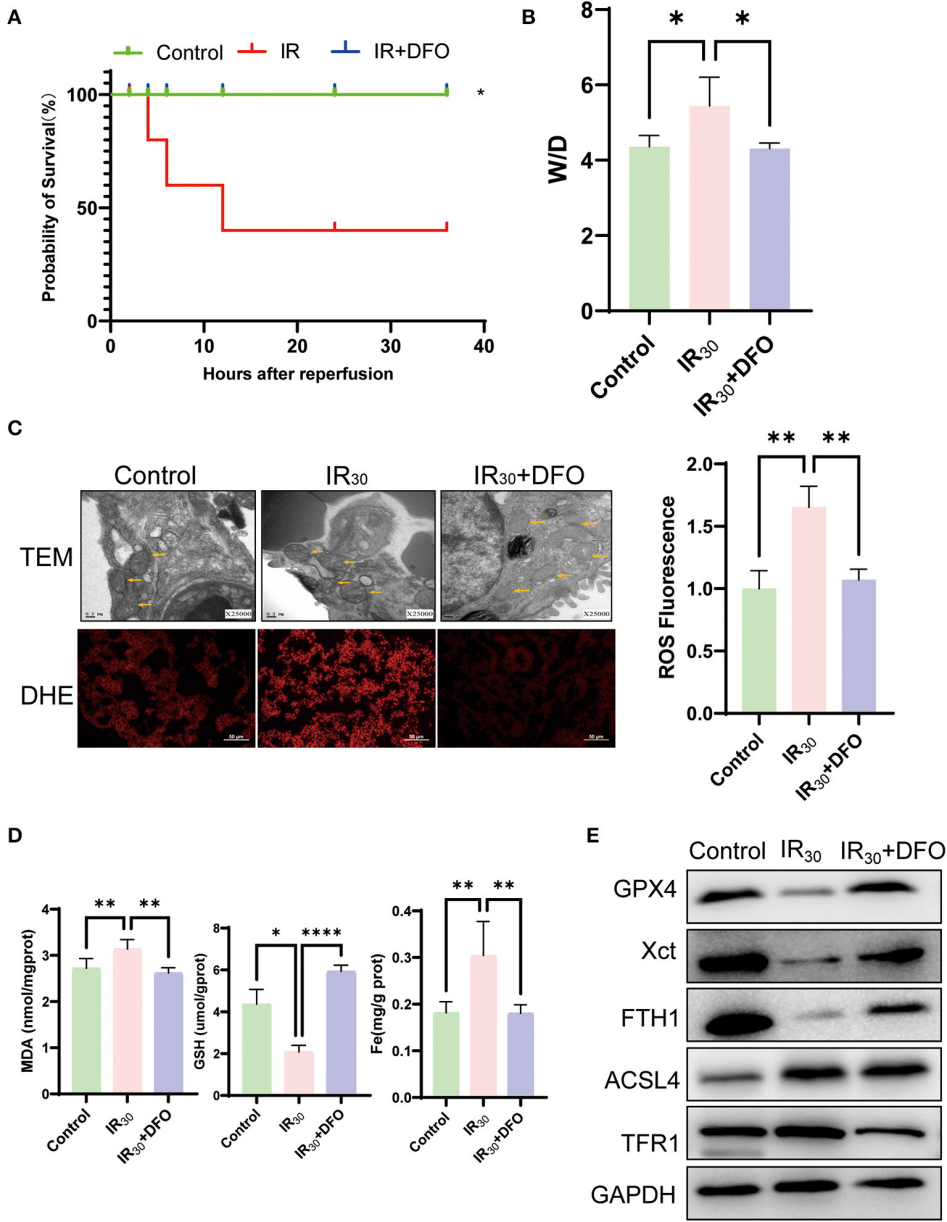
DFO attenuates lung damage by inhibiting ferroptosis in LIRI. **(A)** The survival rate of rats after IR operation (*n* = 10/group). **(B)** Wet–dry weight ratio of the lung (*n* = 5/group). **(C)** TEM analysis and DHE staining of the lung (*n* = 3/group), and TEM results were at 25,000 × magnification, and yellow arrows show mitochondria. **(D)** Levels of MDA, GSH, and Fe in the lung tissue (*n* = 5/group). **(E)** Western blot analysis of GPX4, XCT, FTH1, ACSL4, and TFR1 in the lung (*n* = 3/group). Data are expressed as mean ± standard deviation. A one-way ANOVA was used to analyze statistical differences; *P < 0.05, **P < 0.01, and ****P < 0.0001.

The survival experiment has been carried out to further elucidate the significance of ferroptosis in LIRI. As the result shown, the survival rate of the IR group was just 40%, but while using the ferroptosis inhibitor DFO, the survival rate significantly increased to 100%, indicating that the ferroptosis plays an important role in LIRI (Figure 3A). In addition, the lung tissue wet–dry ratio showed that DFO relieves pulmonary edema induced by IR (Figure 3B). Additionally, the results of DHE staining showed that IR resulted in an accumulation of ROS, which could be apparently repressed by DFO (Figure 3C). Meanwhile, TEM results at 25,000 magnification showed that IR resulted in rupture of the outer mitochondrial membrane after reperfusion; however, similar features were not observed with DFO treatment (as yellow arrows in Figure 3C). It is reported that MDA and GSH serve key roles in maintaining the balance of oxidation and reduction. Along with the increase of MDA and Fe induced by IR, a decrease of GSH was observed in LIRI which could be reversed by DFO (Figure 3D). The results of Western blot showed that GPX4, FTH1, and XCT were upregulated while TFR1 and ACSL4 were reduced after DFO treatment (Figure 3E). Taken collectively, these results suggest that the ferroptosis inhibitor DFO significantly attenuates the lung injury through inhibiting ferroptosis.

### 3.4. DFO mitigates lung damage by suppressing inflammation

**Figure 4:**
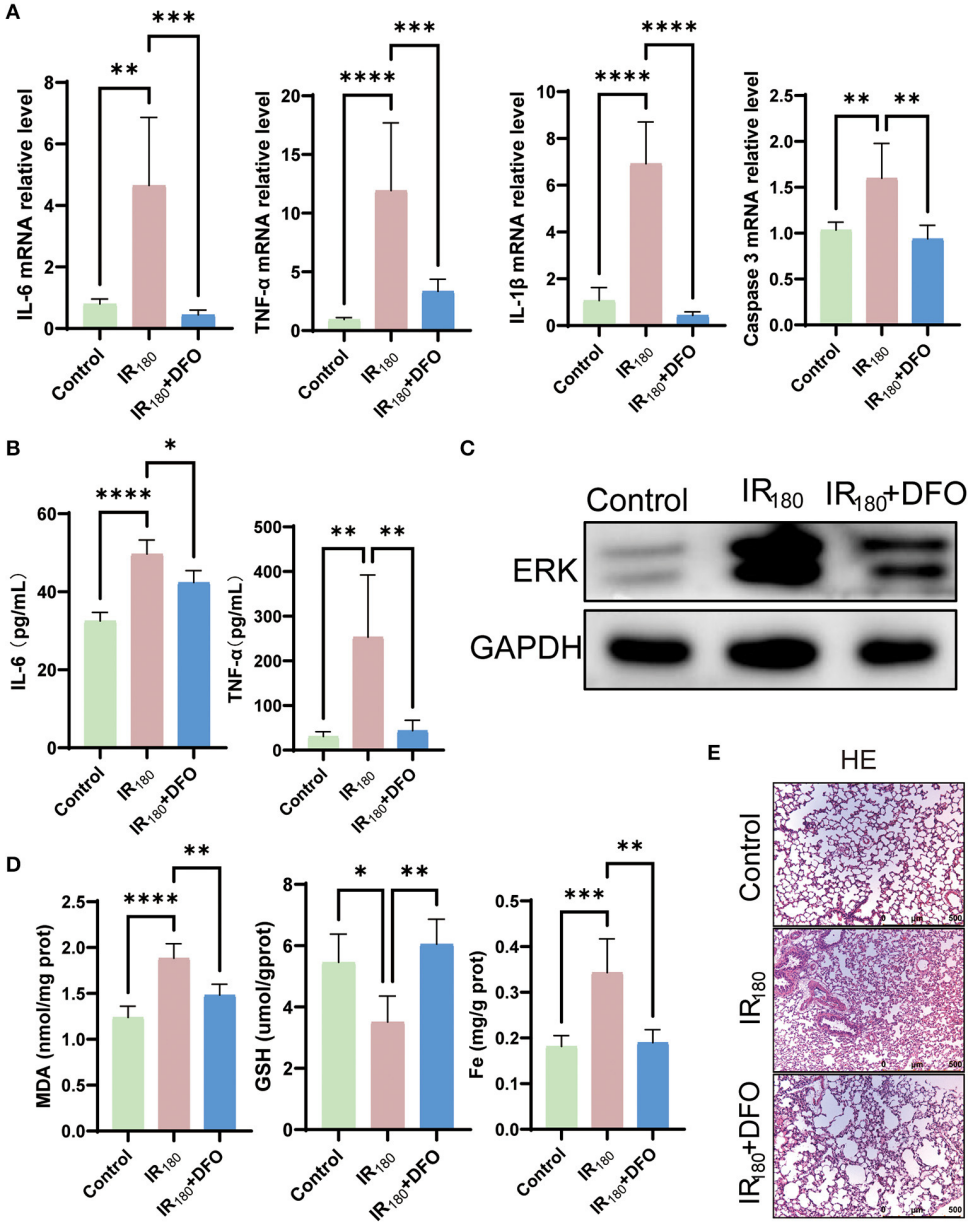
DFO mitigates lung damage by suppressing inflammation. **(A)** qRT-PCR analysis of IL-6, TNF-α, IL-1β, and caspase 3 in the lung (*n* = 5/group). **(B)** ELISA detection of IL-6 and TNF-α in the lung (*n* = 5/group). **(C)** Western blot analysis of total ERK in the lung (*n* = 3/group). **(D)** Levels of MDA, GSH, and Fe in the lung tissue (*n* = 5/group). **(E)** HE staining of the lung (*n* = 3/group). Data are expressed as mean ± standard deviation. A one-way ANOVA was used to analyze statistical differences; *P < 0.05, **P < 0.01, ***P < 0.001, and ****P < 0.0001.

To investigate whether ferroptosis signals contribute to inflammation, the levels of inflammation cytokines were examined after 180 min of reperfusion in LIRI. The results of qRT-PCR analysis showed that DFO dramatically inhibited the levels of IL-6, TNF-α, IL-1β, and caspase 3 (Figure 4A). Moreover, ELISA detection of IL-6 and TNF-α reconfirmed that DFO can suppress inflammation, suggesting that ferroptosis triggers excessive inflammation in LIRI (Figure 4B). Expectedly, the increased total protein level of ERK was inhibited at the 180-min reperfusion phase (Figure 4C). In addition, MDA, GSH, and Fe levels were improved by DFO (Figure 4D). HE staining also showed that lung damage was relieved after DFO treatment (Figure 4E). Therefore, the results above imply that ferroptosis triggers inflammation and the use of ferroptosis inhibitors can suppress inflammation to mitigate lung damage.

## 4. Discussion

Given that ferroptosis and inflammation are increasingly reported to play significant roles in acute lung injury pathogenesis (24, 25), however, the link of ferroptosis to inflammation in LIRI is still unclear and needs to further explore the possible mechanisms. It has been reported that DFO, targeting iron excess, which was widely utilized to inhibit ferroptosis in animal research, can significantly mitigate renal injury by restraining inflammation (26). Our study found that ferroptosis was activated by IR in the early stage of reperfusion, and inhibition of ferroptosis can mitigate lung damage by depressing excessive inflammation.

Herein, we showed the time variation of ferroptosis in LIRI, which is almost compatible with a previous study of intestinal ischemia–reperfusion (27). By controlling the level of cellular iron and the state of lipid peroxidation, numerous substances took part in ferroptosis. It has been suggested that ACSL4, a key enzyme involved in the creation of phospholipids that contain polyunsaturated fatty acids, contributes to lipid peroxidation and the ensuing ferroptosis (8). Increased ACSL4 was captured beginning at the 30-min reperfusion point in the current study, declaring that the lung cells were undergoing ferroptosis. In addition, it is well-known that GPX4, a core gene against ferroptosis through lipid hydroperoxides clearance, is influenced by the GSH level in its activity (28). The generation of GSH can be impacted by the glutamate/cysteine antiporter solute carrier family 7 member 11 (SLC7A11), which can influence cysteine absorption (29). Consistently, the reduction of SLC7A11 and GSH, leading to a weakened GPX4 level, was observed, which reconfirmed that ferroptosis happened in the early stage of reperfusion in LIRI. Inhibiting ferroptosis has been proposed as a potential target for several disorders since it is linked to numerous pathophysiological processes. In this study, GPX4 was shown to be significantly decreased after reperfusion of 30 min, verified by the level of GSH and XCT. The transient induction of GPX4 at 60 min of reperfusion observed in this study may be induced by the transient profit under a period of compensation response. The decreased GPX4 expression was found in prolonged reperfusion, similarly to other anti-ferroptotic factors FTH1 and XCT. However, the complete impact of ferroptosis on the pathogenesis of LIRI presumably is more complicated, and further research is warranted.

In recent years, accumulating studies demonstrate that inflammation is directly associated with iron and lipid metabolism, which could be triggered by various acute injuries in both humans and animals (30, 31). Ferroptosis is, therefore, believed to play a pathogenic role in LIRI, combining oxidative stress and inflammatory responses linked to iron and lipid metabolism. The well-known ferroptosis biomarker COX-2 accelerates the metabolism of AA and increases inflammation by secreting inflammatory signaling molecules (32), which implies that ferroptosis links inflammation through the activation of cyclooxygenase (33). Moreover, it has been reported that hepatic ferroptosis plays an important role as the trigger for initiating inflammation in non-alcoholic steatohepatitis, showing that the expressions of TNF-α, IL-6, and IL-1β were significantly upregulated following ferroptosis (34). Additionally, evidence exhibited that ferroptosis inhibitors can block inflammatory responses in psoriasis with the reduced production of cytokines including TNF-α, IL-6, IL-1α, IL-1β, IL-17, IL-22, and IL-23 (35). In our study, the inflammatory cytokines, including TNF-α, IL-6, and IL-1β, were also found to be greatly increased in the beginning at the 60-min reperfusion points; however, this increase could be inhibited during DFO administration, which revealed a close connection to the initial ferroptosis.

The ERK signal pathway has been well-researched in various acute injuries covering LIRI (20, 36, 37). As a previous study evidenced, ERK expression was significantly suppressed to attenuate the IR-induced lung injury during the administration of preconditioning anti-vascular endothelial growth factor antibody, which suggests that ERK plays a vital role in LIRI, meaning that there must be a complicated link between ERK and ferroptosis (38). It has been reported that ERK could regulate ferritinophagy and inflammation, contributing to ferroptosis in macrophage (39). However, in the current study, ferroptosis was found to be activated by IR in the early stage of reperfusion, while increased ERK was observed at the following reperfusion. Interestingly, the increased ERK can be inhibited after DFO treatment, revealing that ERK might be triggered by ferroptosis. In brief, the interaction between ERK and ferroptosis needs further exploration.

## 5. Conclusion

In this study, we established a LIRI model to explore the underlying mechanism. The relationship between inflammation and ferroptosis was evaluated through the related indicators, showing that the pro-ferroptotic markers COX-2 and ACSL4 were upregulated and anti-ferroptotic genes GPX4, XCT, and FTH1 were depressed. Additionally, the level of IL-6, TNF-α, and IL-1β, especially ERK, were observed to be increased following ferroptosis, suggesting that ferroptosis is activated by IR in the early reperfusion stage, which is the initiator of subsequent inflammation in LIRI. Furthermore, DFO significantly ameliorated lung injury, including improvement in type II alveolar cells' ultrastructure and reactive oxygen species (ROS) production, accompanied by a substantial reduction in the levels of IL-6, TNF-α, and IL-1β, which implied that the TNF-α/IL-6-ERK pathway is the crucial role to lung damage. Note that inhibition of ferroptosis with DFO can depress inflammation and thus attenuate LIRI, which can provide the clinical value for various ferroptosis-related diseases. However, this study only employed male rats, which has limitations, and more experiments need to be carried out in future.

## Data availability statement

The original contributions presented in the study are included in the article/Supplementary material, further inquiries can be directed to the corresponding author.

## Ethics statement

The animal study was reviewed and approved by Experimental Animal Ethics Committee of Wenzhou Medical University.

## Author contributions

WW and FW designed the study. XL and BP analyzed and interpreted data, generated figures and tables, and drafted the manuscript with XiaW. JX and XinW contributed to manuscript drafting. ZS and EZ reviewed and corrected the manuscript. All authors contributed to the article and approved the submitted version.
